# Ability to Generate Patient-Derived Breast Cancer Xenografts Is Enhanced in Chemoresistant Disease and Predicts Poor Patient Outcomes

**DOI:** 10.1371/journal.pone.0136851

**Published:** 2015-09-01

**Authors:** Priscilla F. McAuliffe, Kurt W. Evans, Argun Akcakanat, Ken Chen, Xiaofeng Zheng, Hao Zhao, Agda Karina Eterovic, Takafumi Sangai, Ashley M. Holder, Chandeshwar Sharma, Huiqin Chen, Kim-Anh Do, Emily Tarco, Mihai Gagea, Katherine A. Naff, Aysegul Sahin, Asha S. Multani, Dalliah M. Black, Elizabeth A. Mittendorf, Isabelle Bedrosian, Gordon B. Mills, Ana Maria Gonzalez-Angulo, Funda Meric-Bernstam

**Affiliations:** 1 Department of Surgical Oncology, The University of Texas MD Anderson Cancer Center, Houston, TX, United States of America; 2 Department of Investigational Cancer Therapeutics, The University of Texas MD Anderson Cancer Center, Houston, TX, United States of America; 3 Department of Bioinformatics and Computational Science, The University of Texas MD Anderson Cancer Center, Houston, TX, United States of America; 4 Department of Systems Biology, The University of Texas MD Anderson Cancer Center, Houston, TX, United States of America; 5 Department of Biostatistics, The University of Texas MD Anderson Cancer Center, Houston, TX, United States of America; 6 Department of Veterinary Medicine and Surgery, The University of Texas MD Anderson Cancer Center, Houston, TX, United States of America; 7 Department of Pathology, The University of Texas MD Anderson Cancer Center, Houston, TX, United States of America; 8 Department of Genetics, The University of Texas MD Anderson Cancer Center, Houston, TX, United States of America; 9 Department of Breast Medical Oncology, The University of Texas MD Anderson Cancer Center, Houston, TX, United States of America; University of North Carolina School of Medicine, UNITED STATES

## Abstract

**Background:**

Breast cancer patients who are resistant to neoadjuvant chemotherapy (NeoCT) have a poor prognosis. There is a pressing need to develop *in vivo* models of chemo resistant tumors to test novel therapeutics. We hypothesized that patient-derived breast cancer xenografts (BCXs) from chemo- naïve and chemotherapy-exposed tumors can provide high fidelity *in vivo* models for chemoresistant breast cancers.

**Methods:**

Patient tumors and BCXs were characterized with short tandem repeat DNA fingerprinting, reverse phase protein arrays, molecular inversion probe arrays, and next generation sequencing.

**Results:**

Forty-eight breast cancers (24 post-chemotherapy, 24 chemo-naïve) were implanted and 13 BCXs were established (27%). BCX engraftment was higher in TNBC compared to hormone-receptor positive cancer (53.8% vs. 15.6%, p = 0.02), in tumors from patients who received NeoCT (41.7% vs. 8.3%, p = 0.02), and in patients who had progressive disease on NeoCT (85.7% vs. 29.4%, p = 0.02). Twelve patients developed metastases after surgery; in five, BCXs developed before distant relapse. Patients whose tumors developed BCXs had a lower recurrence-free survival (p = 0.015) and overall survival (p<0.001). Genomic losses and gains could be detected in the BCX, and three models demonstrated a transformation to induce mouse tumors. However, overall, somatic mutation profiles including potential drivers were maintained upon implantation and serial passaging. One BCX model was cultured in vitro and re-implanted, maintaining its genomic profile.

**Conclusions:**

BCXs can be established from clinically aggressive breast cancers, especially in TNBC patients with poor response to NeoCT. Future studies will determine the potential of *in vivo* models for identification of genotype-phenotype correlations and individualization of treatment.

## Introduction

Neoadjuvant chemotherapy (NeoCT) is the standard of care for patients with locally advanced and inflammatory breast cancer, and it is increasingly being used in operable breast cancer both to decrease extent of surgery, and as an *in vivo* indicator of sensitivity to systemic therapy. Ten to 30% of patients will not respond to NeoCT, while 3% of patients have tumor progression while on therapy [1]. Patients with tumor progression during chemotherapy or with significant residual cancer burden after NeoCT are at high risk of relapse [1–3]. There is a pressing need to understand mechanisms of resistance and to identify novel therapeutics that are effective in patients who do not respond to standard NeoCT as well as in those with significant residual disease after NeoCT.

Triple negative breast cancers (TNBC) lack the expression of estrogen, progesterone and HER2 receptors and are not sensitive to estrogen and HER2-targeted therapies. Patients with TNBC have significantly higher pathologic complete response (pCR) rates with chemotherapy compared with non-TNBC [4] and if pCR is achieved, patients with TNBC and non-TNBC have similar survival. In contrast, TNBC patients with residual disease (RD) after chemotherapy have worse overall survival compared with non-TNBC. Therefore there is an urgent need to identify novel therapies for chemoresistant TNBC.

Currently, *in vivo* models available to test novel agents are limited. Cell lines may diverge from their tumors of origin during adaptation to *in vitro* growth conditions and when they are injected to form xenografts. Responses to treatments in cell lines often are not concordant with those observed in patients. Genetically engineered mice can demonstrate specific aspects of oncogenesis, but do not recapitulate the heterogeneity of clinical tumors and rely on the assumption that the same genetic alterations transform both mouse and human cells [5].

Recently there has been great interest in developing patient-derived xenografts (PDXs) from several tumor types including colon cancer, lung cancer, ovarian cancer, head and neck tumors, leukemia as well as breast cancer [6–17]. Hidalgo et al. described an *in vivo* pancreatic cancer patient-derived serial transplantation model and initially reported an engraftment rate of 80%, and a “take”-rate of passagable from the tumors of 93% [18–20]. On expansion of their series, they reported an engraftment rate of 61%, and that engraftment was greater in tumors derived from patients with worse prognosis [21]. Further, a pilot clinical trial suggested a potential role for preclinical testing in PDXs to individualize patient therapy [22].

We hypothesized that tumors from breast cancer patients can be used to establish patient-derived breast cancer xenografts (BCXs) with high fidelity to their original tumors, providing *in vivo* models for TNBC and other chemoresistant tumors. Such a model would require relative stability in the characteristics of the tumor as it is passaged through several generations of immunodeficient mice. To test this hypothesis, we transplanted chemo-naïve breast tumors or residual breast cancers after NeoCT. We determined feasibility of generating passagable BCXs, from residual tumors after NeoCT, and the relative stability of the genomic profile of the patients’ tumors with that of serial generations of BCXs.

## Materials and Methods

### Ethics statement

The research involving human participants was approved by the MD Anderson Institutional Review Board. All patients signed informed consent prior to participation (IRB protocol # LAB07-0950). All animal studies were approved by the MD Anderson Animal Care and Use Committee (ACUF protocol # 02-08-00833). This study was carried out in strict accordance with the recommendations in the Guide for the Care and Use of Laboratory Animals of the National Institutes of Health.

### Patient population

Patients with histologically-confirmed invasive breast cancer were recruited for this study. All patients had greater than 1 cm tumor on physical exam or imaging prior to surgery. Fifty-five patients were consented and underwent breast-conserving surgery or mastectomy for local therapy with sentinel node biopsy with or without axillary lymph node dissection (ALND) for regional therapy; one patient had contralateral lymph node recurrence and had an ALND alone; one had bilateral breast cancer. Three patients underwent surgery in the presence of known distant metastases.

From the first fifty-five breast cancer patients enrolled in the study, eight patients were excluded based on lack of adequate tumor on gross pathology assessment, or hematoxylin and eosin (H&E) staining showed no viable tumor tissue in the donor specimen. Therefore, 48 viable tumors were implanted from 47 patients (one with bilateral breast cancer).

### *In vivo* tumor implantation and histopathologic analysis

The tumor specimen was placed in sterile tissue-culture medium on ice and brought immediately to the animal facility. Tumor was implanted into five 6-week old female BALB/c *nu/nu* mice under isoflurane anesthesia, and all efforts were made to minimize suffering. Two ~0.3 cm skin incisions were made with subcutaneous pockets on the left and right mid-back. One tumor piece (~1–3 mm) was inserted into each pocket and the skin was closed. When tumor diameter reached 1.5 cm, mice were euthanized, tumors were excised, cut into ~1x1x1 mm fragments, and passaged to successive generations of 5 mice. At each passage, remaining tumor was snap frozen in liquid nitrogen and stored at –80°C. Samples of these tumors were fixed in formalin and embedded in paraffin blocks. All primary tumors and BCX samples were assessed by H&E staining for morphology. In selected samples immunohistochemistry for CD20, CD3, CD45, and cytokeratin was performed in the Department of Veterinary Medicine and Surgery and Department of Pathology.

### *In vitro* growth of conditionally reprogrammed cells and reimplantation

A 1.5 cm diameter BCX-010 xenograft was excised from the mouse and finely minced in F-media as described in Liu et al. [23]. Tumor fragments were digested for 4 hr at 37°C in F-media using collagenase/hyaluronidase (Stemcell Technologies; 1X) with occasional light mixing. The slurry was filtered, pelleted, washed, and plated in F-media supplemented with 10 μM Y-27632 (Enzo). The media was changed after 24 hours. Once the culture reached ~70–90% confluence, the cells were split at a ~1:4 ratio. After 4 passages, the cells were collected and 1x10^8^ cells were subcutaneously injected with or without 50% Matrigel into the flank of athymic nude (MD Anderson) or CIEA NOG (Taconic) mice. STR fingerprinting was used to confirm the human/BCX-010 origin of the cultured cells and the single cell-based xenografts.

### DNA isolation and DNA fingerprinting

Genomic DNA was isolated from frozen or FFPE tumor tissue using the QIAamp DNA Mini Kit (Qiagen). Short tandem repeat (STR) DNA fingerprinting was performed with the AmpFℓSTR Identifiler kit (Applied Biosystems) at the Characterized Cell Line Core Facility.

### Polymerase chain reaction

Genomic DNA was amplified using Herculase II DNA polymerase (Agilent). Primer sequences were: Zfp42 (mouse) (5’-TGAGATTAGCCCCGAGACTGAG-3’ and 5’-CGTCCCCTTTGTCATGTACTCC-3’), HBB (5’-CCTGAGGAGAAGTCTGCCGTTA-3’ and 5’-GAACCTCTGGGTCCAAGGGTAG) [24], PTEN (5’-GGAATCCAGTGTTTCTTTTAAATACC-3’ and 5’-TCCAGGAAGAGGAAAGGAAAA-3’), and RB1 (5’-TCCCATGGATTCTGAATGTG-3’ and 5’-CGTTGTGCACATGTACCCTAGA-3’).

### Fluorescence in situ hybridization

FISH was performed at the Molecular Cytogenetics Core Facility. The all mouse centromere probe (Kreatech, Netherlands) or the human centromere 16 probe (Abbott Molecular) was applied and slides were stained with DAPI.

### Copy number and somatic mutation determination

Genomic DNA from five of the patients (1, 2, 4, 5, and 6) and corresponding P3 BCXs were submitted to Affymetrix for molecular inversion probe (MIP) assay with copy number and somatic mutation determination. The Affymetrix platform interrogated 300,008 single nucleotide polymorphisms and 412 somatic mutations in 45 genes. The MIP assay was performed as previously described [25].

Single nucleotide polymorphism (SNP) genotyping was done at the Genomics Core Facility. Hot spot mutation testing was performed in 46 cancer related genes including *PIK3CA* on pre-treatment and on-treatment biopsies (at 12 weeks of chemotherapy) using the Ion Ampliseq Cancer Panel (Life Technologies) to assess hotspot mutations as previously described [26]

Next generation targeted exome sequencing was performed as previously described [27, 28]. For genomic profiling, DNA from mouse normal tissue, and six patients’ normal tissue or normal blood, primary tumors and corresponding BCXs: passage 0 (P0), P1, P4, P7, P10, P13, and P16 of BCX-006, -010, -011, and P0 and P1 of BCX-017, -022, -024 were evaluated as well as P6 of BCX-022, and P7 of BCX-024 [13, 15, 24–28].

Briefly library prep was performed using KAPA library prep kit (Kapa Biosystems, Inc), and equimolar amounts of DNA were pooled (8–12 samples per pool) for capture of 201 genes that are clinically relevant in cancer (S1 Table). The captured libraries were sequenced on a HiSeq 2000 (Illumina Inc.). In addition, whole exome sequencing was performed for normal tissue or blood, primary tumors and corresponding P0 and P1 for BCX-010, -017, -022, and -024. For data analysis, reads were aligned to human reference assembly hg19 using BWA software and duplicated reads were removed using samtools. Single nucleotide variants (SNVs) and small indels were called using VarScan2 [29].

### Functional proteomics

Reverse phase protein array (RPPA) was performed as previously described [30] to compare the proteomic profile of P0 patient tumors and corresponding BCXs (P1, P2 and P3), in models BCX-001, 002, 004, 005, and 006. A logarithmic value reflecting the relative amount of each protein in each sample was generated. The median polish normalized RPPA data set consisted of 154 proteins.

### Statistical analysis

Descriptive statistics were used to describe engraftment rates. BCX success rates in different tumor types and clinical scenarios were compared using Fisher’s Exact 2-tailed analysis. Linear mixed effects (LME) model was used to assess the differences in passage time among passage numbers. The LME model included fixed effect of passage number (1 vs. 2 vs. 3) and random effect of model.

For RPPA, unsupervised clustering was applied to all tumor samples across all of the proteins, using Spearman Correlation and complete linkage. Paired t-test was performed to compare changes in relative protein amounts between, P0, P1, and P3. The p values were fitted to a-beta-uniform mixture-BUM model to calculate the corresponding false discovery rate (FDR) [31–33]. FDR <0.2 was considered significant [34]. To compare protein expression levels across multiple generations, a g-statistic was used [35]. R packages including GeneCycle, q value and ClassComparison were employed [34].

## Results

### Ability to generate patient-derived breast cancer xenografts is enhanced in TNBC and in chemoresistant disease

Forty-eight viable tumors were implanted from 47 patients: one patient had a bilateral tumor implanted as separate models. Implants were followed for 6 months after implantation. The resultant BCXs were designated MDA-BCX-001 to -055 (MD Anderson Breast Cancer Xenograft 001 to 055). At the time of diagnosis, the median age of patients was 55 years (range, 29–86). At presentation, 32 tumors were hormone receptor-positive (HR+), 13 were TNBC, and 3 were HER2-positive (HER2+). At presentation, average tumor size (± standard deviation) was 4.3±3.1 cm (range, 0.8–15). Twenty-four of the tumors (50%), came from patients who received NeoCT. Of the 23 patients who had NeoCT with intact primary tumors, 7 had progressive disease, 7 had a partial response, and 9 had stable disease. One patient had an axillary cross-metastasis and that responded to therapy.

**Fig 1:**
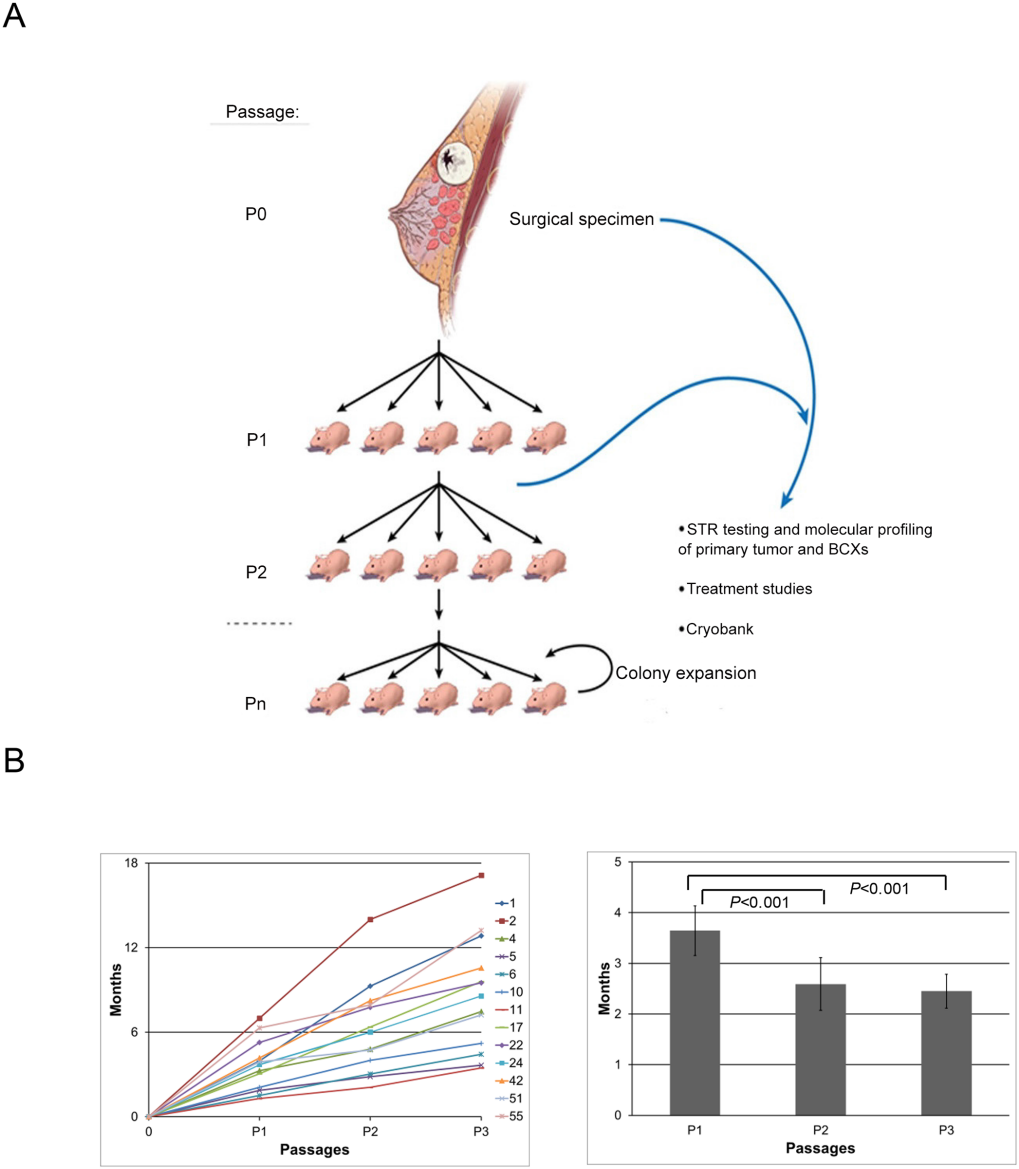
Generating and maintaining patient-derived breast cancer xenografts (BCXs) and their time to passage. (A) After surgery, patient tumors (P0) were implanted into nude mice, creating a patient-derived BCX, passage 1 (P1). When tumors reached 1.5 cm diameter, they were harvested and implanted into 5 new mice (P2), and subsequent passages respectively. Patient tumors and BCXs were evaluated by STR and selected passages underwent molecular and histologic characterization. (B) Y-axis depicts time to reach 1.5 cm with each passage. The graph shows thirteen BCXs that were serially passage. (C) Time to passage (in months) at from implantation to first passage (P1), P1 to P2 (P2), and P1 to P3 (P3). Time to passage at P2 and P3 were compared to time to passage at from implantation to first passage (P1).

Tumor from the surgical specimens (designated as “P0”) was implanted (Fig 1A) and mice with tumor growth at the site of implantation were considered to have successful engraftment. Tumors derived from 13 of the 48 specimens (27%) were successfully engrafted.

BCXs that reached 1.5 cm in size were passaged to another set of mice (P2; Fig 1A). Time to tumor size of 1.5 cm diameter (i.e. passage from P1 to P2) was an average of 2.6 months (range 0.8–7 months; median 1.9 months) (Fig 1B). P1 tumors were passaged to P2 generation and then were transferred into the third passage (P3) in all 13 models. When the time from implantation to first passage (P1) was compared to the time from first passage to second passage (P1-P2 interval) or to the time from second passage to third passage (P2-P3 interval), was significantly longer than both latter passages (p<0.001 for both; Fig 1C). Table 1 demonstrates comparison of patient and tumor characteristics and success of BCX engraftment. BCX engraftment was higher in TNBC compared to HR+ tumors (7 of 13, 53.8% vs. 5 of 32, 15.6%, p = 0.02). One of three HER2+ tumors which had received NeoCT engrafted.

**Table 1:** Tumor characteristics and engraftment rate.

		Implanted (N)	Passaged (N, %)	P Value
	TNBC	13	7 (53.8%)	0.02*
Breast Cancer Subtype	HER2+	3	1 (33.3%)	
	HR+	32	5 (15.6%)	
	TNBC	9	5 (55.5%)	0.02**
Yes	HER2+	2	1 (50%)	
	HR+	13	5 (38.5%)	
NeoCT	TNBC	4	2 (50%)	
No	HER2+	1	0 (0%)	
	HR+	19	0 (0%)	
	Progressive Disease	7	6 (85.7%)	0.02***
Response to NeoCT	Stable Disease	9	2 (22.2%)	
	Partial Response	8	3 (37.5%)	

NeoCT: Neoadjuvant chemotherapy

*P value by Fisher exact analysis, comparing BCX engraftment in TNBC to HR+ breast cancer.

**P value by Fisher exact analysis, comparing BCX engraftment in NeoCT to no NeoCT implantations.

***P value by Fisher exact analysis, comparing BCX engraftment in tumors that progressed on NeoCT vs. those that had stable disease/partial response.

Engraftment was higher for tumors that had been exposed to NeoCT (10 of 24, 41.7% vs. 2 of 24, 8.3%, p = 0.02). Further, of the 7 patients who had had progressive disease on NeoCT, tumor implantation from 6 patients (85.7%) developed passagable BCXs; in contrast, tumors from 5 (29.4%) of 17 patients who had stable disease or partial response to NeoCT lead to passagable BCXs (p = 0.02).

Of note, two TNBC BCX models were derived from tumors not classified as TNBC prior to chemotherapy. For the BCX-011 model, the pre-NeoCT core biopsy was ER+, but the post-NeoCT and the emerging BCX-011 P1 samples were ER-. In addition, the pre-NeoCT core biopsy was HER2+ and ER+ for BCX-017, but the surgical sample after neoadjuvant chemotherapy was ER+, HER2- and the emerging BCX-017 P1 sample was TNBC. Notably this latter patient received paclitaxel with concomitant trastuzumab followed by 5-FU, epirubicin, and cyclophosphamide with concomitant trastuzumab.

**Fig 2:**
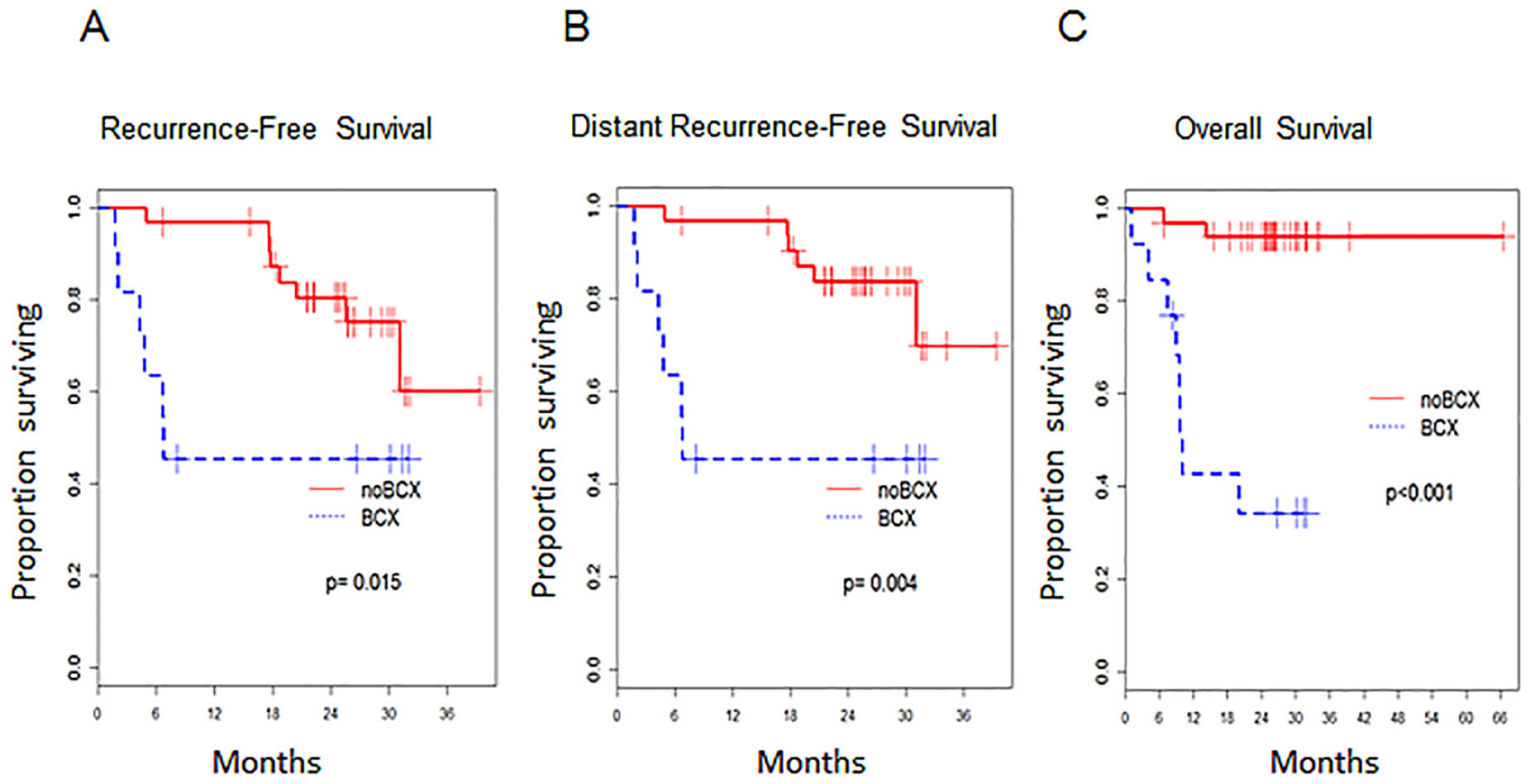
Survival Outcomes in Patients Based on BCX development. (A) Recurrence-free survival (in months) in patients whose tumors developed BCX versus those did not (no BCX). (B) Distant recurrence-free survival (in months) in patients whose tumors developed BCX versus those did not (no BCX). (C) Overall survival (in months) in patients whose tumors developed BCX versus those did not (no BCX).

At a median follow-up of 25 months (range, 1.8–39.4) after surgery, 12 of 44 patients with unilateral Stage II or III disease at surgery developed distant metastasis; the patient with bilateral tumors was disease-free at last follow up. For 5 patients, passagable tumors were derived prior to their development of distant metastases (Table 2). Among the 44 patients with unilateral localized disease, patients whose tumors developed BCXs had significantly lower recurrence-free survival (p = 0.015; Fig 2A) and distant recurrence-free survival (p = 0.004; Fig 2B) compared to patients whose tumors did not develop BCXs. Three patients with Stage IV disease underwent surgery; BCXs developed from the tumors of two patients. When all 47 patients are considered, patients whose tumors developed BCXs had significantly lower overall survival (p<0.001, Fig 2C).

**Table 2:** Disease progression and engrafted BCXs.

	Metastasis-free survival (months)	Overall survival (months)**	Implant to latest follow-up (months)	Engraftment (N, %)***	P0→P1 (months)
**BCX-001**	6.7	8.5	8.5	2/5 (40%)	4
**BCX-002**	0*	7.4	7.4	2/5 (40%)	7
**BCX-004**	4.8	9.5	9.5	2/5 (40%)	3.3
**BCX-005**	6.8	20.0	20	5/5 (100%)	1.9
**BCX-006**	4.3	9.6	9.6	5/5 (100%)	1.5
**BCX-010**	0*	1	1	3/5 (60%)	2.1
**BCX-011**	2.1	4.1	4.1	2/5 (40%)	1.3
**BCX-017**	Met free	Alive	32.0	5/5 (100%)	3.1
**BCX-022**	Met free	Alive	30.1	5/5 (100%)	5.3
**BCX-024**	Met free	Alive	31.4	4/5 (80%)	3.7
**BCX-042**	Met free	Alive	20.5	3/5 (60%)	4.2
**BCX-051**	1.8	10.0	10.0	3/5 (60%)	3.3
**BCX-055**	Met free	Alive	8.2	1/5 (20%)	6.3

P0→P1 is time from implantation of patient tumor into mice until the tumor reaches 1.5 cm at which time they were transplanted into the next group of 5 nude mice.

*These patients presented with metastatic disease.

**Overall survival from surgery.

***Numbers and percentage of tumor bearing mice after implantation in a group of 5 mice.

### DNA fingerprinting demonstrated donor origin of patient-derived xenografts

STR DNA fingerprinting was used on all models that were serially transplantable to evaluate specific regions (loci) within nuclear DNA, to confirm that the xenografts were patient-derived. The STR profiles of the 13 passagable BCX models were all unique, showing no matches to known DNA fingerprints in American Type Culture Collection, the Deutsche Sammlung von Mikroorganismen und Zellkulturen, Cell Line Integrated Molecular Authentication, MD Anderson Cancer Center databases or to each other. The P0 genomic DNA from each patient resulted in a match with the corresponding BCX specimens for these 13 models (BCX-001, 002, 004, 005, 006, 010, 011, 017, 022, 024, 042, 051, and 055).

### Histopathologic and molecular characterization identified murine origin tumors in two mice

A breast pathologist and a veterinary pathologist compared H&E stained slides of surgical specimens and BCXs. For BCX models designated successful BCX models, no histologic differences were found. The tumor derived from Patient 3 took the longest time (8.4 months) to develop a 1.5 cm mass. STR characterization of P0 demonstrated a unique human tumor. However, STR from P1 showed lack of amplification with seven of eight STR primer sets and discordance with P0 in the only one primer set that was successfully amplified. Mouse implanted with tumor from Patient 8 established a tumor; time from P0 to P1 and P1 to P2 took 5.2 and 4.5 months, respectively. However, tumor growth stopped at P2. STR from P1 showed lack of amplification. Therefore, STR results raised concern that both BCX-003 and -008 series xenografts may not be of human origin.

Although STR characterization of P0, P1 and P3 confirmed that BCX-001 series was indeed derived from Patient 1, histologic evaluation of late passages of the BCX-001 revealed morphologically undifferentiated tumors that lost the characteristics of epithelial/breast tumor cells, raising concerns about the fidelity of the model.

To determine whether the BCXs were of human origin, we performed PCR using two sets of primers designed to amplify a target sequence in which human (HBB) and murine (Zfp42) DNA could be distinguished (S2A Fig) [24]. We tested serial passages collected from BCX-001 and BCX-003 models as well as BCX-004 as a control. As expected, all three patient tumors (P0) showed no amplification of murine DNA, but amplification was seen with human primers (S2A Fig). In BCX-004, the mouse sequence was detected in P1 and P3, but at significantly less intensity than the human DNA product; this may be attributable to murine stroma and leukocytes within the xenograft. In contrast, for BCX-003, PCR of DNA from P1 and P3 xenografts demonstrated only amplification with murine DNA primers, suggesting the tumors were solely of murine origin. For BCX-001, there predominantly was amplification of human DNA at early passages including P4, but at P5, there was an abrupt loss of human DNA and amplification of murine DNA exclusively. Interestingly, this corresponded to an increase in growth rate (S2B Fig). The other model, BCX-008 P0 and P1 were tested similarly. Human sequence was detected in P0, and only mouse sequence detected in P1 (data not shown).

When we examined H&E stained slides of BCX-001, passages 1 to 4, showed well differentiated epithelial tumors, whereas passages 5 to 8 showed tumors composed of dense neoplastic cells with smaller round nuclei and minimal basophilic cytoplasm suggestive of spontaneous murine lymphoma (S2C Fig). However, immunohistochemistry for CD20, CD3, CD45 and cytokeratin were negative on P5, suggesting this is an undifferentiated tumor of unknown tissue origin. FISH using mouse and human centromere probes demonstrated presence of human and mouse DNA in P1 but only mouse DNA in the later passage, P6 (S2D Fig). For BCX-003, the H&E stained slide of P1 showed areas of normal mouse mammary glands adjacent to mammary adenocarcinoma (S2E Fig). FISH revealed only mouse DNA in P1 (S2E Fig). This confirms a spontaneous mammary adenocarcinoma of mouse origin rather than human.

### Functional proteomics demonstrated alterations in PI3K signaling

**Fig 3:**
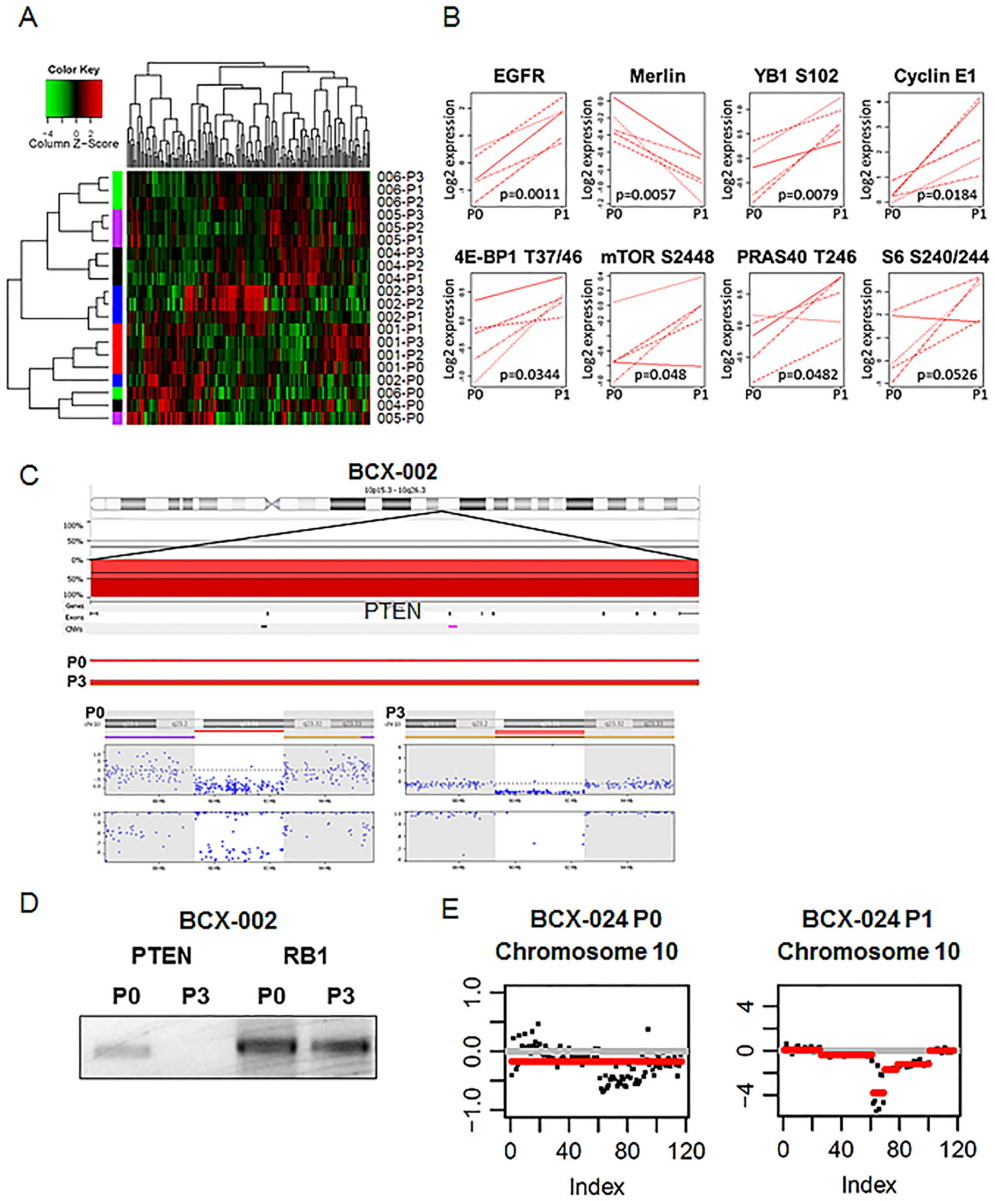
Molecular differences between patients’ tumors and BCXs. (A) Unsupervised clustering of proteomic profile of patient tumors and BCXs as determined by RPPA. Each protein tested represents a column: Red, high expression; green, low expression. Samples are listed on the right side. Left, cluster trees of sample groups; top, cluster trees of proteins. Each BCX model’s P1-3 generation clustered together, demonstrating relative stability of the proteomic profile once growth in mouse is established. However, all P0 generations clustered together, suggesting that differences between patient tumor-xenograft proteomic profiles is greater than inter-tumoral differences. (B) Selected proteins and phosphoproteins that are differentially expressed between patient tumors (P0) and the first-generation of BCXs passaged through nude mice (P1). Protein levels were compared between the two groups with RPPA; all shown have a FDR 0.1 or less. (C) Copy number analysis determined PTEN loss in MDA-BCX-002. Top panel shows the chromosome 10 ideogram and the PTEN gene. Deletions are plotted in red below the 0% baseline, and dark red indicates homozygous loss. The lowest portion of the top panel separates out P0 and P3. A heterozygous PTEN loss is detected in P0, and in the P3, the second PTEN allele is lost, resulting in a homozygous PTEN loss. Bottom panel shows the PTEN gene ideogram followed by the copy number aberration plot and the allele frequency plot for P0 and P3. A heterozygous PTEN loss is detected in P0 (single red line), and a homozygous PTEN loss is detected in P3 (double red line). Each blue dot corresponds to an individual probe on the array. The brown and purple lines mark the thresholds for loss of heterozygosity (LOH) and allelic imbalance regions, respectively. (D) PCR confirmed PTEN deletion in genomic DNA from Patient 2 tumor and BCX-002 P3. PTEN was undetectable in P3 but present in P0. RB1, another tumor suppressor gene, is detected in both samples and included for comparison. (E) PTEN loss demonstrated by next generation sequencing in BCX-024. P0 on the left, P1 on the right.

Unsupervised hierarchical clustering of RPPA was used to evaluate the functional proteomic profile of five of the ten patient’s surgical specimens and corresponding serial transplanted *in vivo* tumors. 135 unique proteins and phosphoproteins were evaluated; 19 were done in duplicate, 2 in triplicate as part of the quality control process. Upon unsupervised clustering, patient tumors clustered together, separate from the BCXs, suggesting that there are significant differences between the primary tumor and the BCXs (Fig 3A). However, each BCX lineage clustered together demonstrated that their proteomic profile remains relatively stable for several *in vivo* passages.

When protein expression was compared between the original breast tumors (P0) versus those from the first BCX passage (P1), levels of 103 of 154 proteins were differentially expressed at a FDR of 0.2 (S2 Table). Eight of these proteins are displayed in Fig 3B: EGFR, merlin, YB1 S102, Cyclin E1, 4E-BP1 T37/46, mTOR S2448, PRAS40 T246 and S6 S240/244. Importantly, a number of these proteins are part of the same PI3K pathway indicative either of increased pathway activity or alternatively of decreased human stromal content. All patient tumor-BCX differences remained significant when the P0 tumors were compared to both P1 and P2 and the first three transplants (P1, P2 and P3).

### Copy number analysis determined loss of PTEN in two BCX models

MIP genotyping assays detect genetic alterations including candidate point mutations between specimens and are particularly well designed for copy number analysis [36]. A total of 412 somatic mutations in 45 genes were analyzed in genomic DNA extracted from the surgical specimens from patients 1, 2, 4, 5, and 6 and compared to corresponding BCXs. A heterozygous loss of an allele of PTEN was identified in the P0 tumor derived from patient 2. In the corresponding P3 of BCX-002, the second PTEN allele was lost, resulting in a homozygous PTEN loss (Fig 3C). Consistent with this finding, when PCR was performed with primers to human PTEN, PTEN genomic DNA was detected from P0 DNA but not from DNA extracted from P3 BCXs (Fig 3D). However, another interpretation could be that human stroma PTEN is present in P0 and that the loss of human stroma allows a homozygous PTEN loss that was present to begin with to be identified.

Targeted exome sequencing using a 201 gene platform was performed in six models: BCX-006, -010, -011, -017, -022, and -024 [27]. Copy number based on targeted exome sequencing demonstrated loss of PTEN in P1 the BCX-024 model (Fig 3E and S3 Table). Loss of PTEN in BCX-024 P1 was also confirmed by whole exome sequencing (S4 Table).

### Genomic sequencing demonstrated relative genomic stability of somatic mutations in BCX models

**Fig 4:**
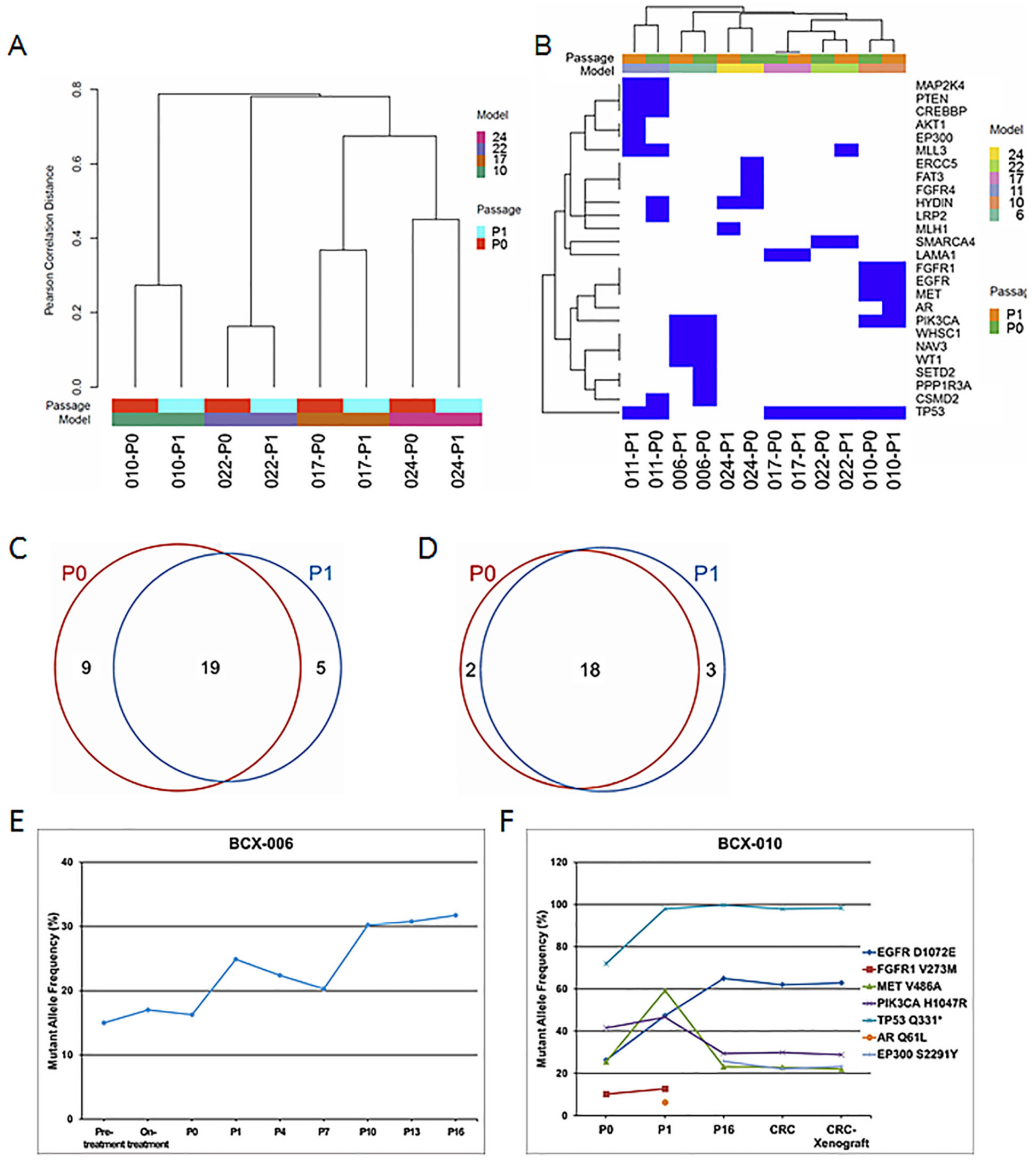
Analysis of mutation data. (A) Clustering of whole exome sequencing mutation data based on the mutation status of the genes in P0 and P1 samples of four models. (B) Clustering of targeted exome sequencing data based on the mutation status of 201 genes in P0 and P1 samples of six models. (C) Venn diagram of mutations in P0 and P1 samples of six models on the targeted exome sequencing platform. (D) Venn diagram of high allelic frequency mutations (10% or higher) on targeted exome sequencing in P0 and P1 samples of six models. Allele frequency cutoff was 10%. (E) Genomic stability of *PIK3CA* mutation in BCX-006 model. Fine needle aspiration biopsy samples of before and after 12 weeks of neoadjuvant chemotherapy (including a rapalog) were sequenced by ion torrent. Patient’s P0 tumor and BCX-006 P1 tumor and subsequent passages were analyzed by targeted exome sequencing. *PIK3CA* H1047R allele frequencies are presented. (F) Conditionally reprogrammed cells (CRC) derived from BCX-010 were passaged four times *in vitro* and then injected into mice flanks. The cultured cells and CRC-derived xenografts maintained a mutation profile similar to the originating PDX.

Whole exome sequencing was performed in the mouse host DNA as well as the matched P0-P1—normal host DNA in 4 models: BCX-010, -017, -022, and -024 (S4 and S5 Tables). Unsupervised clustering clustered each BCX P1 with the P0 parental tumor (Fig 4A).

As described above, targeted exome sequencing was performed in 6 models: BCX-006, -010, -011, -017, -022, and -024. The mutations detected are listed in S6 Table. Upon unsupervised clustering each BCX clustered with its parental tumor (Fig 4B). Of 28 mutations detected on targeted exome sequencing in P0 tumors, 19 (68%) were preserved in P1 tumors (Fig 4C). Of 21 high allelic frequency mutations (allelic mutation frequency 10% or higher) detected in P1, 18 (86%) were also detected in the parental tumors (Fig 4D). In five of the models, serial passages were sequenced. With serial passaging, mutations were preserved, including potential driver mutations such as *PIK3CA* mutations, but additional alterations were observed in some models. In the BCX-006 model, pretreatment and on-treatment biopsies obtained during NeoCT with a rapalog-containing combination therapy regimen were also available. These demonstrated that the *PIK3CA* mutation was detected at a relatively high allelic frequency in the pretreatment and 12 week samples as well as in the P0 sample, and was preserved upon multiple BCX passages (Fig 4E). Although the allelic frequency increased in BCX passages, this was accompanied by an increase in mutant allelic frequency of other mutations as well, suggesting that this may be attributable to increase in tumor cellularity (S5 Table). Thus there did not appear to be selection of the mutation during therapy.

### Somatic mutation profile is retained with *in vitro* culture and implantation

An experimental challenge is experimentally manipulating the PDX cells for functional target validation. Thus we also explored the possibility of developing conditionally reprogrammed cells (CRC) from PDXs and, and the effect of serial transplantation and *in vivo* re-implantation using the rapidly growing BCX-010 model. We cultured and expanded cells from BCX-010 [23]. After 4 passages, we injected these BCX-010 cultured cells into two strains of immune-deficient mice (*nu*/*nu* and NOG) with and without Matrigel. Rapidly growing tumors developed in all cases. STR fingerprinting confirmed identity of the CRC and the CRC-derived xenograft. The cultured cells as well as the CRC-derived xenograft maintained a mutation profile very similar to the originating PDX (Fig 4F).

## Discussion

PDXs have considerable promise for drug testing as they contain heterogeneity that is absent in cell line-derived xenografts [37]. They represent high-fidelity models for research and potentially even for individual patient treatment planning. In our study, we have demonstrated a good engraftment rate and rate in initiating passagable BCXs from chemotherapy-resistant breast cancers. Indeed, the take rate in chemotherapy treated BCX was increased compared to treatment naïve tumors consistent with the enrichment of stem cell-like cells in chemotherapy treated tumor [38]. We did observe modest alterations in the molecular profile, including a potential loss of *PTEN*, and activation of PI3K/mTOR signaling on functional proteomics. We also identified a previously under-reported problem, that of murine transformation in three models. However, the overall genomic profile of BCXs was relatively stable suggesting that they have the potential to function as useful *in vivo* models.

Engraftment efficiency among PDXs varies in the literature. For example, engraftment rate for pancreatic cancers has been at over 60% [21], whereas for breast cancers at 12–50%, with approximately 5–21% achieving serial transplants [5, 11, 12, 17, 39]. The disparity in transplantation efficiency is likely influenced by patient tumor type and host mouse strain [13], but it may also be a reflection of the aggressive biology of donor tumors [14, 15]. A comparison of the patient tumors that formed and that did not form xenografts identified upregulation of cell cycle, cell signaling and cytoskeleton pathways, but downregulation of immune response in successfully formed xenografts [40]. In our hands, the tumor take rate was significantly higher from TBNC patients, and patients who had received NeoCT. Of note we actively enrolled chemoresistant patients; progressive disease on NeoCT is seen in only 3% of patients and is associated with a significantly worse prognosis [1]. Strikingly BCXs were successfully developed in six of seven patients who had progressive disease on chemotherapy. Of the patients in whom we established models, two already had distant metastases at the time of surgery, and six developed metastases within an average of 4.4 months. This is consistent with reports that the ability to successfully engraft correlates with tumor aggressiveness [41]. This is further supported in our study with the finding that patients whose tumors developed BCXs had significantly lower recurrence-free survival, distant recurrence-free survival and overall survival.

It has been proposed that PDXs may hold promise to prioritize treatment options in patients with advanced disease [22, 42]. Some studies have reported preliminary data correlating antitumor efficacy in patient derived xenograft models with patient outcomes [6]. It is notable that in several patients we were able to develop serially transplantable tumors from patients prior to the patients’ development of distant relapse. This timeline would allow us to explore the utility of these models for *in vivo* testing (AKA as avatar models).

It is important to determine if serially passaged xenografts remain representative of the original patient tumors. In previous series of pancreatic, breast, and colorectal cancer xenografts, no major variations between primary tumors and xenografts had been found [6, 9, 10, 14, 19, 41, 43]. However, Ding et al., identified genetic variation between a primary basal-like breast cancer and the corresponding metastatic tumor, as well as a serially transplanted xenograft from the same patient [44]. In our series both the genomic and proteomic profiles were, for the most part, preserved between the primary tumors and BCXs.

With three BCX implantations we observed development of a mouse tumor. The possibility of inducing a host transformation has been reported in cell line-derived xenografts in the immunocompromised host [45, 46]. Further, others have also reported lymphoproliferative tumors of patient origin [16]. Thus, we recommend that once a PDX is established, H&E, and STR should be performed to confirm the fidelity of histopathologic characteristics and to confirm a unique human model that matches the patient tumor is established, respectively. Further, key molecular features should be intermittently re-assessed.

Comparing the primary tumor with the BCXs, we did observe increased activity of the PI3K/Akt/mTOR pathway. This pathway has been implicated in breast cancer development and progression. In our study, we did identify a change in the amount of loss of PTEN in one tumor during adaptation to growth in mice, however as indicated below whether this represents, loss, selection or a change in the amount of human stroma present has not been determined. On RPPA, we also demonstrated several alterations in PI3K signaling consistent with increased PI3K pathway activity in BCXs. Cancer cells evolve and acquire a more resistant phenotype with progression. Recently several studies, including our own, demonstrated a discrepancy in molecular markers between primary tumors and metastases [26, 47–49]. Models to delve into mechanisms of molecular evolution are lacking. Our studies demonstrate that there may be some molecular evolution upon *in vivo* implantation and upon subsequent passaging. It is possible that PI3K pathway activation may give BCXs a growth or survival advantage, leading to accumulation of, or selection for PI3K pathway aberrations. One explanation of our findings is clonal selection [8, 11, 44]. It is unclear whether the PTEN changes we observed represents enrichment already present in a small subpopulation of cells-clones-in the original patient tumor, or whether it was an acquired alteration under selection pressure for growth in the murine environment. However alternate explanation of our findings may be technical, such as enrichment for tumor DNA in BCX-002 serial passages with the loss of human stroma (with normal PTEN copy number, which would be detected on the MIP arrays) [37, 50] and replacement with mouse stroma (with a mouse PTEN DNA which would not be detected by to the MIP arrays) [51]. Similarly increase in tumor cellularity in the BCX may result in an appearance of increased PI3K activity in the overall tumor specimen without a change in the tumor component. The apparent increase in PI3K activity could also represent difference in sample handling and conservation of phosphorylation specific events. There are differences in post-resection processing of primary tumors and BCXs; primary tumors are inked and potentially radiographed for margin analysis and only then specimens retrieved for research. In contrast BCXs are immediately frozen after harvesting; this difference could contribute to apparent higher protein phosphorylation in BCX. This is especially important while assessing PI3K signaling as we have already shown that there are differences in several phospho-residues between matched primary tumor core biopsies and surgical samples obtained after similar processing [30]. This is also consistent with the finding that once BCXs are established, there functional proteomic profiles remained stable, with passages of each BCX model clustering together on unsupervised clustering. Further work is needed to define which of the changes between P0 and P1 represent tumor evolution, and whether BCXs can be used to better delineate the interplay between tumor heterogeneity, drug response, and molecular evolution.

## Conclusions

We have developed an *in vivo* breast cancer model with primary breast tumors from patients with significant residual disease or progression after NST who are at high risk of distant relapse. Their chemoresistant tumors were used to create serially transplantable BCXs. Some genetic and proteomic changes were identified in the process of engraftment and serial passage, especially aberrations in the PI3K/mTOR signaling pathway. However, the majority of the genomic alterations were retained. Future work will determine whether these changes recapitulate molecular evolution that occurs with tumor progression and metastases. This model holds promise as a novel platform to discover molecular aberrations that can be targeted for therapy in chemoresistant disease.

## Supporting Information

S1 Fig Determination of human versus mouse origin of BCXs and characterization of lineage. **A.** Two sets of primers were used to amplify a target sequence [Zfp42, mouse (M) and HBB, human (H)] from (P0) and subsequent BCX (P1, etc.) from Patients 4, 3, and 1. As expected, amplification of the mouse sequence was absent in all patient tumors (P0). For Patient 4, but not for Patient 3, human DNA bands were preserved in the BCX. For BCX-001, human DNA was amplified in early passages but there was loss of human DNA in P5 onwards. **B.** BCXs derived from Patient 1’s tumor were passaged from the surgical specimen (P0) to subsequent generations. Graph shows time to passage of tumor to the next generation when tumors reach 1.5 cm in diameter, and demonstrates a distinct increase in growth speed beginning with P5. **C.** H&E stained sections of Patient 1-derived xenograft at various passages: P0 –breast carcinoma of patient; P3 and P4 passages show well-differentiated epithelial tumors; P5 and P7 passages show undifferentiated neoplastic cells with smaller round nuclei and minimal cytoplasm. Original magnification, 400x. **D.** FISH using fluorescent labeled mouse centromere probe (red) and human centromere probe (green) on BCX-001 at early (P1) and later (P6) generations. P1 is positive only for human probe and P6 is positive only for mouse probe. Original magnification, 100x. **E.** Characterization of the BCX-003 P1 xenograft. H&E stained sections of P1 tumor show mouse mammary adenocarcinoma (middle panel) developed within the mouse mammary gland tissue (left panel) at the site of tumor engraftment. Rectangle in the middle panel represents the area captured in FISH (right panel). Original magnification, 400x (left and middle panels). FISH demonstrates positive red fluorescence of tumor and connective tissue for mouse centromere probe and negative green fluorescence for human centromere probe. Original magnification, 100x.
(TIF)

S1 Table List of genes assessed by targeted exome sequencing. (DOCX)

S2 Table Differentially expressed proteins between P0 and P1. (XLSX)

S3 Table Copy number alterations detected in all tested passages of six models by targeted exome sequencing (BCX-006, -010, -011, 017, -022, -024). High amplification cutoff was ≥4 (highlighted in red) and high deletion cutoff was ≤1 (highlighted in green).
(XLSX)

S4 Table Copy number alterations detected in P0 and P1 of four models by whole exome sequencing (BCX-010, -017, -022, -024). High amplification cutoff was ≥4 (highlighted in red) and high deletion cutoff was ≤1 (highlighted in green).
(XLSX)

S5 Table Somatic mutations detected in P0 and P1 of four models by whole exome sequencing (BCX-010, -017, -022, -024). (XLSX)

S6 Table Somatic mutations detected in all tested passages of six models by targeted exome sequencing (BCX-006, -010, -011, 017, -022, -024). (XLSX)

## References

[1] Caudle AS, Gonzalez-Angulo AM, Hunt KK, Liu P, Pusztai L, Symmans WF, et al Predictors of tumor progression during neoadjuvant chemotherapy in breast cancer. J Clin Oncol. 2010;28(11):1821–8. Epub 2010/03/17. doi: JCO.2009.25.3286 [pii] 10.1200/JCO.2009.25.3286 20231683 PMC2860366

[2] Kuerer HM, Newman LA, Smith TL, Ames FC, Hunt KK, Dhingra K, et al Clinical course of breast cancer patients with complete pathologic primary tumor and axillary lymph node response to doxorubicin-based neoadjuvant chemotherapy. J Clin Oncol. 1999;17(2):460–9. Epub 1999/03/18. . 10080586 10.1200/JCO.1999.17.2.460

[3] Symmans WF, Peintinger F, Hatzis C, Rajan R, Kuerer H, Valero V, et al Measurement of residual breast cancer burden to predict survival after neoadjuvant chemotherapy. J Clin Oncol. 2007;25(28):4414–22. Epub 2007/09/06. doi: JCO.2007.10.6823 [pii] 10.1200/JCO.2007.10.6823 . 17785706

[4] Liedtke C, Mazouni C, Hess KR, Andre F, Tordai A, Mejia JA, et al Response to neoadjuvant therapy and long-term survival in patients with triple-negative breast cancer. J Clin Oncol. 2008;26(8):1275–81. 10.1200/JCO.2007.14.4147 . 18250347

[5] Vargo-Gogola T, Rosen JM. Modelling breast cancer: one size does not fit all. Nature reviews. 2007;7(9):659–72. Epub 2007/08/28. doi: nrc2193 [pii] 10.1038/nrc2193 . 17721431

[6] Ilie M, Nunes M, Blot L, Hofman V, Long-Mira E, Butori C, et al Setting up a wide panel of patient-derived tumor xenografts of non-small cell lung cancer by improving the preanalytical steps. Cancer medicine. 2015;4(2):201–11. 10.1002/cam4.357 25470237 PMC4329004

[7] Cottu P, Marangoni E, Assayag F, de Cremoux P, Vincent-Salomon A, Guyader C, et al Modeling of response to endocrine therapy in a panel of human luminal breast cancer xenografts. Breast cancer research and treatment. 2012;133(2):595–606. Epub 2011/10/18. 10.1007/s10549-011-1815-5 . 22002565

[8] Vick B, Schneider S, Ksienzyk B, Greif PA, Fiegl M, Subklewe M, et al Genetic Profiling By Targeted, Deep Resequencing Confirms That a Murine Xenograft Model Of Acute Myeloid Leukemia (AML) Recapitulates The Mutational Landscape Of The Human Disease and Provides Evidence For Clonal Heterogeneity and Clonal Evolution. Blood. 2013;122(21):49.

[9] Uronis JM, Osada T, McCall S, Yang XY, Mantyh C, Morse MA, et al Histological and molecular evaluation of patient-derived colorectal cancer explants. PLoS ONE. 2012;7(6):e38422 Epub 2012/06/08. 10.1371/journal.pone.0038422 22675560 PMC3366969

[10] Hao C, Wang L, Peng S, Cao M, Li H, Hu J, et al Gene mutations in primary tumors and corresponding patient-derived xenografts derived from non-small cell lung cancer. Cancer letters. 2015;357(1):179–85. 10.1016/j.canlet.2014.11.024 25444907 PMC4301580

[11] Bergamaschi A, Hjortland GO, Triulzi T, Sorlie T, Johnsen H, Ree AH, et al Molecular profiling and characterization of luminal-like and basal-like in vivo breast cancer xenograft models. Molecular oncology. 2009;3(5–6):469–82. Epub 2009/08/29. 10.1016/j.molonc.2009.07.003 . 19713161 PMC5527532

[12] Zhang X, Claerhout S, Prat A, Dobrolecki LE, Petrovic I, Lai Q, et al A renewable tissue resource of phenotypically stable, biologically and ethnically diverse, patient-derived human breast cancer xenograft models. Cancer research. 2013;73(15):4885–97. 10.1158/0008-5472.CAN-12-4081 23737486 PMC3732575

[13] Morton CL, Houghton PJ. Establishment of human tumor xenografts in immunodeficient mice. Nature protocols. 2007;2(2):247–50. Epub 2007/04/05. doi: nprot.2007.25 [pii] 10.1038/nprot.2007.25 . 17406581

[14] Marangoni E, Vincent-Salomon A, Auger N, Degeorges A, Assayag F, de Cremoux P, et al A new model of patient tumor-derived breast cancer xenografts for preclinical assays. Clin Cancer Res. 2007;13(13):3989–98. Epub 2007/07/04. doi: 13/13/3989 [pii] 10.1158/1078-0432.CCR-07-0078 . 17606733

[15] Weroha SJ, Becker MA, Enderica-Gonzalez S, Harrington SC, Oberg AL, Maurer MJ, et al Tumorgrafts as in vivo surrogates for women with ovarian cancer. Clin Cancer Res. 2014;20(5):1288–97. 10.1158/1078-0432.CCR-13-2611 24398046 PMC3947430

[16] Klinghammer K, Raguse JD, Plath T, Albers AE, Joehrens K, Zakarneh A, et al A comprehensively characterized large panel of head and neck cancer patient-derived xenografts identifies the mTOR inhibitor everolimus as potential new treatment option. International journal of cancer. 2015;136(12):2940–8. 10.1002/ijc.29344 . 25404014

[17] Garber K. From human to mouse and back: 'tumorgraft' models surge in popularity. Journal of the National Cancer Institute. 2009;101(1):6–8. Epub 2009/01/01. doi: djn481 [pii] 10.1093/jnci/djn481 . 19116380

[18] Lin MT, Tseng LH, Kamiyama H, Kamiyama M, Lim P, Hidalgo M, et al Quantifying the relative amount of mouse and human DNA in cancer xenografts using species-specific variation in gene length. BioTechniques. 2010;48(3):211–8. Epub 2010/04/03. doi: 000113363 [pii] 10.2144/000113363 20359302 PMC3065779

[19] Rubio-Viqueira B, Jimeno A, Cusatis G, Zhang X, Iacobuzio-Donahue C, Karikari C, et al An in vivo platform for translational drug development in pancreatic cancer. Clin Cancer Res. 2006;12(15):4652–61. Epub 2006/08/11. doi: 12/15/4652 [pii] 10.1158/1078-0432.CCR-06-0113 . 16899615

[20] Rajeshkumar NV, De Oliveira E, Ottenhof N, Watters J, Brooks D, Demuth T, et al MK-1775, a potent Wee1 inhibitor, synergizes with gemcitabine to achieve tumor regressions, selectively in p53-deficient pancreatic cancer xenografts. Clin Cancer Res. 2011;17(9):2799–806. Epub 2011/03/11. doi: 1078-0432.CCR-10-2580 [pii] 10.1158/1078-0432.CCR-10-2580 21389100 PMC3307341

[21] Garrido-Laguna I, Uson M, Rajeshkumar NV, Tan AC, de Oliveira E, Karikari C, et al Tumor engraftment in nude mice and enrichment in stroma- related gene pathways predict poor survival and resistance to gemcitabine in patients with pancreatic cancer. Clin Cancer Res. 2011;17(17):5793–800. Epub 2011/07/12. doi: 1078-0432.CCR-11-0341 [pii] 10.1158/1078-0432.CCR-11-0341 21742805 PMC3210576

[22] Hidalgo M, Bruckheimer E, Rajeshkumar NV, Garrido-Laguna I, De Oliveira E, Rubio-Viqueira B, et al A pilot clinical study of treatment guided by personalized tumorgrafts in patients with advanced cancer. Molecular cancer therapeutics. 2011;10(8):1311–6. Epub 2011/06/16. doi: 1535-7163.MCT-11-0233 [pii] 10.1158/1535-7163.MCT-11-0233 . 21673092 PMC4629061

[23] Liu X, Ory V, Chapman S, Yuan H, Albanese C, Kallakury B, et al ROCK inhibitor and feeder cells induce the conditional reprogramming of epithelial cells. The American journal of pathology. 2012;180(2):599–607. 10.1016/j.ajpath.2011.10.036 22189618 PMC3349876

[24] Ryba T, Battaglia D, Pope BD, Hiratani I, Gilbert DM. Genome-scale analysis of replication timing: from bench to bioinformatics. Nature protocols. 2011;6(6):870–95. Epub 2011/06/04. doi: 10.1038/nprot.2011.328 nprot.2011.328 [pii]. 21637205 10.1038/nprot.2011.328 PMC3111951

[25] Wang Y, Moorhead M, Karlin-Neumann G, Wang NJ, Ireland J, Lin S, et al Analysis of molecular inversion probe performance for allele copy number determination. Genome biology. 2007;8(11):R246. Epub 2007/11/22. doi: gb-2007-8-11-r246 [pii] 10.1186/gb-2007-8-11-r246 18028543 PMC2258201

[26] Gonzalez-Angulo AM, Ferrer-Lozano J, Stemke-Hale K, Sahin A, Liu S, Barrera JA, et al PI3K pathway mutations and PTEN levels in primary and metastatic breast cancer. Molecular cancer therapeutics. 2011;10(6):1093–101. Epub 2011/04/15. doi: 1535-7163.MCT-10-1089 [pii] 10.1158/1535-7163.MCT-10-1089 21490305 PMC3112276

[27] Chen K, Meric-Bernstam F, Zhao H, Zhang Q, Ezzeddine N, Tang LY, et al Clinical Actionability Enhanced through Deep Targeted Sequencing of Solid Tumors. Clin Chem. 2015;61(3):544–53. 10.1373/clinchem.2014.231100 . 25626406 PMC4511273

[28] Hassan B, Akcakanat A, Holder AM, Meric-Bernstam F. Targeting the PI3-kinase/Akt/mTOR signaling pathway. Surgical oncology clinics of North America. 2013;22(4):641–64. Epub 2013/09/10. 10.1016/j.soc.2013.06.008 . 24012393 PMC3811932

[29] Koboldt DC, Zhang Q, Larson DE, Shen D, McLellan MD, Lin L, et al VarScan 2: somatic mutation and copy number alteration discovery in cancer by exome sequencing. Genome research. 2012;22(3):568–76. Epub 2012/02/04. 10.1101/gr.129684.111 22300766 PMC3290792

[30] Meric-Bernstam F, Akcakanat A, Chen H, Sahin A, Tarco E, Carkaci S, et al Influence of biospecimen variables on proteomic biomarkers in breast cancer. Clin Cancer Res. 2014;20(14):3870–83. Epub 2014/06/05. 10.1158/1078-0432.CCR-13-1507 24895461 PMC4112583

[31] Pounds S, Morris SW. Estimating the occurrence of false positives and false negatives in microarray studies by approximating and partitioning the empirical distribution of p-values. Bioinformatics (Oxford, England). 2003;19(10):1236–42. Epub 2003/07/02. . 12835267 10.1093/bioinformatics/btg148

[32] Hochberg Y, Benjamini Y. More powerful procedures for multiple significance testing. Statistics in medicine. 1990;9(7):811–8. Epub 1990/07/01. . 2218183 10.1002/sim.4780090710

[33] Efron B, Tibshirani R. Empirical bayes methods and false discovery rates for microarrays. Genetic epidemiology. 2002;23(1):70–86. Epub 2002/07/12. 10.1002/gepi.1124 . 12112249

[34] Ring BZ, Chang S, Ring LW, Seitz RS, Ross DT. Gene expression patterns within cell lines are predictive of chemosensitivity. BMC genomics. 2008;9:74 Epub 2008/02/12. 10.1186/1471-2164-9-74 18261237 PMC2263043

[35] Wichert S, Fokianos K, Strimmer K. Identifying periodically expressed transcripts in microarray time series data. Bioinformatics (Oxford, England). 2004;20(1):5–20. Epub 2003/12/25. . 14693803 10.1093/bioinformatics/btg364

[36] Thompson PA, Brewster AM, Kim-Anh D, Baladandayuthapani V, Broom BM, Edgerton ME, et al Selective genomic copy number imbalances and probability of recurrence in early-stage breast cancer. PLoS ONE. 2011;6(8):e23543 Epub 2011/08/23. 10.1371/journal.pone.0023543 PONE-D-11-02818 [pii]. 21858162 PMC3155554

[37] Julien S, Merino-Trigo A, Lacroix L, Pocard M, Goere D, Mariani P, et al Characterization of a large panel of patient-derived tumor xenografts representing the clinical heterogeneity of human colorectal cancer. Clin Cancer Res. 2012;18(19):5314–28. Epub 2012/07/25. 10.1158/1078-0432.CCR-12-0372 . 22825584

[38] Li X, Lewis MT, Huang J, Gutierrez C, Osborne CK, Wu MF, et al Intrinsic resistance of tumorigenic breast cancer cells to chemotherapy. Journal of the National Cancer Institute. 2008;100(9):672–9. 10.1093/jnci/djn123 . 18445819

[39] Visonneau S, Cesano A, Torosian MH, Miller EJ, Santoli D. Growth characteristics and metastatic properties of human breast cancer xenografts in immunodeficient mice. The American journal of pathology. 1998;152(5):1299–311. Epub 1998/05/20. 9588898 PMC1858587

[40] Monsma DJ, Monks NR, Cherba DM, Dylewski D, Eugster E, Jahn H, et al Genomic characterization of explant tumorgraft models derived from fresh patient tumor tissue. Journal of translational medicine. 2012;10:125 Epub 2012/06/20. 10.1186/1479-5876-10-125 22709571 PMC3439334

[41] DeRose YS, Wang G, Lin YC, Bernard PS, Buys SS, Ebbert MT, et al Tumor grafts derived from women with breast cancer authentically reflect tumor pathology, growth, metastasis and disease outcomes. Nature medicine. 2011;17(11):1514–20. Epub 2011/10/25. 10.1038/nm.2454 22019887 PMC3553601

[42] Morelli MP, Calvo E, Ordonez E, Wick MJ, Viqueira BR, Lopez-Casas PP, et al Prioritizing phase I treatment options through preclinical testing on personalized tumorgraft. J Clin Oncol. 2012;30(4):e45–8. Epub 2011/12/21. doi: JCO.2011.36.9678 [pii] 10.1200/JCO.2011.36.9678 . 22184402 PMC4874230

[43] Hidalgo M, Amant F, Biankin AV, Budinska E, Byrne AT, Caldas C, et al Patient-derived xenograft models: an emerging platform for translational cancer research. Cancer Discov. 2014;4(9):998–1013. 10.1158/2159-8290.CD-14-0001 25185190 PMC4167608

[44] Ding L, Ellis MJ, Li S, Larson DE, Chen K, Wallis JW, et al Genome remodelling in a basal-like breast cancer metastasis and xenograft. Nature. 2010;464(7291):999–1005. Epub 2010/04/16. 10.1038/nature08989 20393555 PMC2872544

[45] Pathak S, Nemeth MA, Multani AS, Thalmann GN, von Eschenbach AC, Chung LW. Can cancer cells transform normal host cells into malignant cells? British journal of cancer. 1997;76(9):1134–8. Epub 1997/01/01. 9365160 10.1038/bjc.1997.524 PMC2228111

[46] Pathak S, Nemeth MA, Multani AS. Human tumor xenografts in nude mice are not always of human origin: a warning signal. Cancer. 1998;83(9):1891–3. Epub 1998/11/07. 10.1002/(SICI)1097-0142(19981101)83:9<1891::AID-CNCR3>3.0.CO;2-U [pii]. . 9806646

[47] Akcakanat A, Sahin A, Shaye AN, Velasco MA, Meric-Bernstam F. Comparison of Akt/mTOR signaling in primary breast tumors and matched distant metastases. Cancer. 2008;112(11):2352–8. 10.1002/cncr.23456 18386830 PMC2819051

[48] Liedtke C, Broglio K, Moulder S, Hsu L, Kau SW, Symmans WF, et al Prognostic impact of discordance between triple-receptor measurements in primary and recurrent breast cancer. Ann Oncol. 2009;20(12):1953–8. Epub 2009/07/15. doi: mdp263 [pii] 10.1093/annonc/mdp263 19596702 PMC2791352

[49] Meric-Bernstam F, Frampton GM, Ferrer-Lozano J, Yelensky R, Perez-Fidalgo JA, Wang Y, et al Concordance of genomic alterations between primary and recurrent breast cancer. Molecular cancer therapeutics. 2014;13(5):1382–9. 10.1158/1535-7163.MCT-13-0482 24608573 PMC4348062

[50] Moro M, Bertolini G, Tortoreto M, Pastorino U, Sozzi G, Roz L. Patient-derived xenografts of non small cell lung cancer: resurgence of an old model for investigation of modern concepts of tailored therapy and cancer stem cells. J Biomed Biotechnol. 2012;2012:568567 Epub 2012/05/02. 10.1155/2012/568567 22547927 PMC3324927

[51] Hylander BL, Punt N, Tang H, Hillman J, Vaughan M, Bshara W, et al Origin of the vasculature supporting growth of primary patient tumor xenografts. Journal of translational medicine. 2013;11:110 Epub 2013/05/04. 10.1186/1479-5876-11-110 23639003 PMC3660244

