# Correction: Ability to Generate Patient-Derived Breast Cancer Xenografts Is Enhanced in Chemoresistant Disease and Predicts Poor Patient Outcomes

**DOI:** 10.1371/journal.pone.0151121

**Published:** 2016-03-08

**Authors:** Priscilla F. McAuliffe, Kurt W. Evans, Argun Akcakanat, Ken Chen, Xiaofeng Zheng, Hao Zhao, Agda Karina Eterovic, Takafumi Sangai, Ashley M. Holder, Chandeshwar Sharma, Huiqin Chen, Kim-Anh Do, Emily Tarco, Mihai Gagea, Katherine A. Naff, Aysegul Sahin, Asha S. Multani, Dalliah M. Black, Elizabeth A. Mittendorf, Isabelle Bedrosian, Gordon B. Mills, Ana Maria Gonzalez-Angulo, Funda Meric-Bernstam

Table 1 appears incorrectly in the published article. Please see the correct Table 1 and its legend below.

**Table 1:** Tumor characteristics and engraftment rate.

		Implanted (N)	Passaged (N, %)	P Value
Breast Cancer Subtype	TNBC	13	7 (53.8%)	0.02*
	HER2+	3	1 (33.3%)	** **
	HR+	32	5 (15.6%)	
NeoCT—Yes	TNBC	9	5 (55.5%)	0.02**
	HER2+	2	1 (50%)	
	HR+	13	5 (38.5%)	
NeoCT—No	TNBC	4	2 (50%)	
	HER2+	1	0 (0%)	
	HR+	19	0 (0%)	
Response to NeoCT	Progressive Disease	7	6 (85.7%)	0.02***
	Stable Disease	9	2 (22.2%)	
	Partial Response	8	3 (37.5%)	

NeoCT: Neoadjuvant chemotherapy

*P value by Fisher exact analysis, comparing BCX engraftment in TNBC to HR+ breast cancer.

**P value by Fisher exact analysis, comparing BCX engraftment in NeoCT to no NeoCT implantations.

***P value by Fisher exact analysis, comparing BCX engraftment in tumors that progressed on NeoCT vs. those that had stable disease/partial response.
