# Supplementary figures and images for: Ability to Generate Patient-Derived Breast Cancer Xenografts Is Enhanced in Chemoresistant Disease and Predicts Poor Patient Outcomes
Source: PLoS One. 2015 Sep 1;10(9):e0136851. doi: 10.1371/journal.pone.0136851 (PMC4556673; doi:10.1371/journal.pone.0136851)

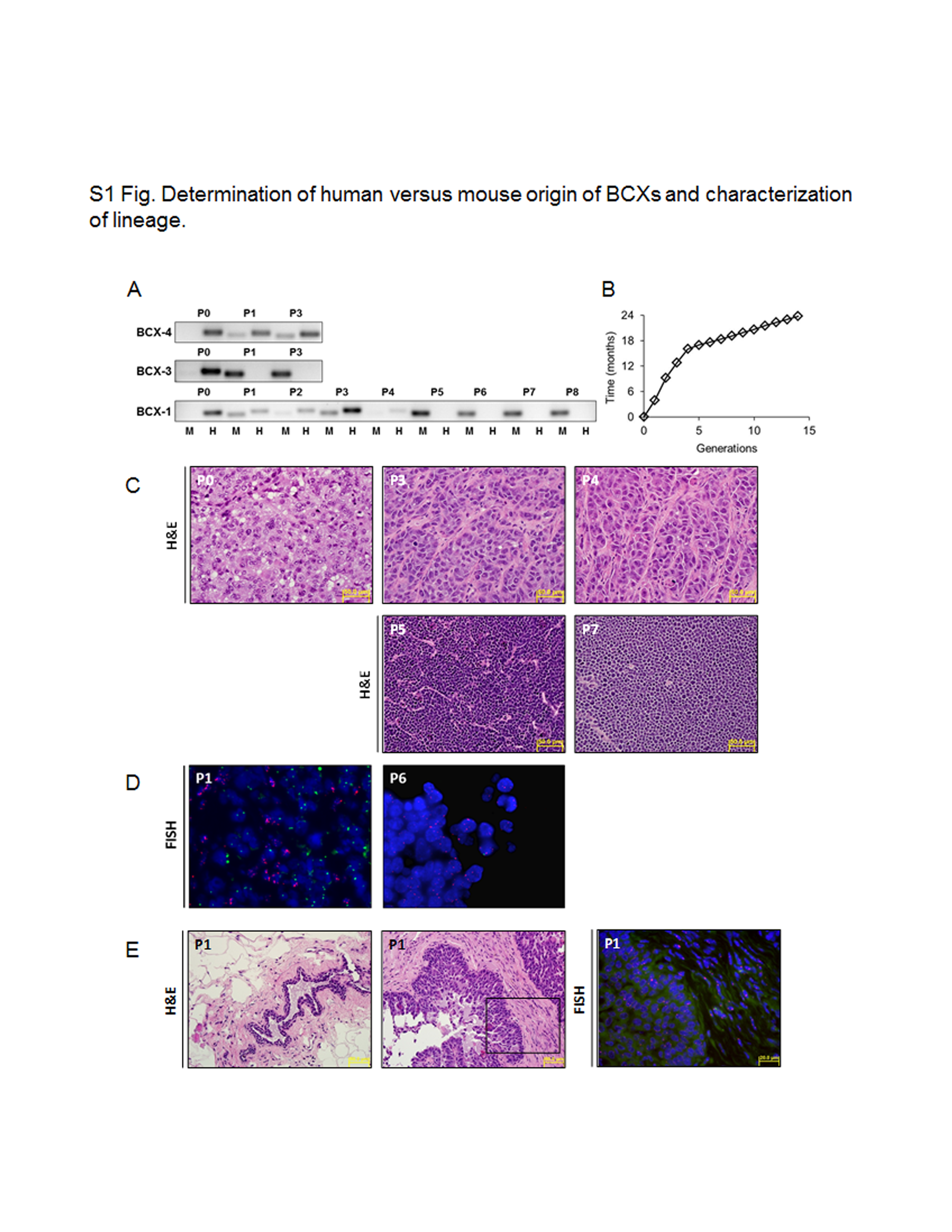

Supplement: S1 Fig — A. Two sets of primers were used to amplify a target sequence [Zfp42, mouse (M) and HBB, human (H)] from (P0) and subsequent BCX (P1, etc.) from Patients 4, 3, and 1. As expected, amplification of the mouse sequence was absent in all patient tumors (P0). For Patient 4, but not for Patient 3, human DNA bands were preserved in the BCX. For BCX-001, human DNA was amplified in early passages but there was loss of human DNA in P5 onwards. B. BCXs derived from Patient 1’s tumor were passaged from the surgical specimen (P0) to subsequent generations. Graph shows time to passage of tumor to the next generation when tumors reach 1.5 cm in diameter, and demonstrates a distinct increase in growth speed beginning with P5. C. H&E stained sections of Patient 1-derived xenograft at various passages: P0 –breast carcinoma of patient; P3 and P4 passages show well-differentiated epithelial tumors; P5 and P7 passages show undifferentiated neoplastic cells with smaller round nuclei and minimal cytoplasm. Original magnification, 400x. D. FISH using fluorescent labeled mouse centromere probe (red) and human centromere probe (green) on BCX-001 at early (P1) and later (P6) generations. P1 is positive only for human probe and P6 is positive only for mouse probe. Original magnification, 100x. E. Characterization of the BCX-003 P1 xenograft. H&E stained sections of P1 tumor show mouse mammary adenocarcinoma (middle panel) developed within the mouse mammary gland tissue (left panel) at the site of tumor engraftment. Rectangle in the middle panel represents the area captured in FISH (right panel). Original magnification, 400x (left and middle panels). FISH demonstrates positive red fluorescence of tumor and connective tissue for mouse centromere probe and negative green fluorescence for human centromere probe. Original magnification, 100x. (TIF) [file pone.0136851.s001.tif]
