# Supplementary material for: Ability to Generate Patient-Derived Breast Cancer Xenografts Is Enhanced in Chemoresistant Disease and Predicts Poor Patient Outcomes
Source: PLoS One. 2015 Sep 1;10(9):e0136851. doi: 10.1371/journal.pone.0136851 (PMC4556673; doi:10.1371/journal.pone.0136851)
Supplement: S1 Table — (DOCX) [file pone.0136851.s002.docx]

S1 Table. List of genes assessed by targeted exome sequencing

| **Symbol** | **Chromossome region** | **Name** |
| --- | --- | --- |
| ABL1 | chr9:133,710,831-133,763,060 | c-abl oncogene 1, non-receptor tyrosine kinase |
| ACVR1B | chr12:52345486-52390859 | activin A receptor, type IB |
| ADAMTS12 | chr5:33,527,287-33,892,124 | ADAM metallopeptidase with thrombospondin type 1 motif, 12 |
| AKAP3 | chr12:4,724,677-4,754,358 | A kinase (PRKA) anchor protein 3 |
| AKT1 | chr14:105235689-105262080 | v-akt murine thymoma viral oncogene homolog 1 |
| ALK | chr2:29415641-30144432 | anaplastic lymphoma kinase (Ki-1) |
| APC | chr5:112073556-112181935 | adenomatous polyposis of the colon gene |
| AR | chrX:66763874-66944119 | androgen receptor |
| ARAF | chrX:47420578-47431319 | v-raf murine sarcoma 3611 viral oncogene homolog |
| ARID1A | chr1:27022522-27108601 | AT rich interactive domain 1A (SWI-like) |
| ASXL1 | chr20:30946153-31027121 | additional sex combs like 1 |
| ATM | chr11:108093559-108239826 | ataxia telangiectasia mutated |
| ATR | chr3:142168078-142297668 | ataxia telangiectasia and Rad3 related |
| ATRX | chrX:76760359-77041719 | alpha thalassemia/mental retardation syndrome X-linked |
| AURKA | chr20:54944445-54967351 | aurora kinas A |
| AURKB | chr17:8108050-8113883 | aurora kinase B |
| BAI3 | chr6:69345632-70099402 | brain-specific angiogenesis inhibitor 3 |
| BAP1 | chr3:52435027-52444009 | BRCA1 associated protein-1 (ubiquitin carboxy-terminal hydrolase) |
| BRAF | chr7:140433815-140624564 | v-raf murine sarcoma viral oncogene homolog B1 |
| BRCA1 | chr17:41196313-41277500 | familial breast/ovarian cancer gene 1 |
| BRCA2 | chr13:32889617-32973809 | familial breast/ovarian cancer gene 2 |
| CARD11 | chr7:2945769-3083579 | caspase recruitment domain family, member 11 |
| CASP8 | chr2:202122754-202152434 | caspase 8, apoptosis-related cysteine peptidase |
| CBL | chr11:119076990-119178858 | Cas-Br-M (murine) ecotropic retroviral transforming sequence |
| CD19 | chr16:28943260-28950661 | CD19 molecule |
| CDH1 | chr16:68771195-68869444 | cadherin 1, type 1, E-cadherin (epithelial) (ECAD) |
| CDH10 | chr5:24487210-24644911 | cadherin 10, type 2 |
| CDH11 | chr16:64980685-65155919 | cyclin-dependent kinase 11 |
| CDK4 | chr12:58142005-58146164 | cyclin-dependent kinase 4 |
| CDK6 | chr7:92234237-92465941 | cyclin-dependent kinase 6 |
| CDKN2A | chr9:21967752-21994490 | cyclin-dependent kinase inhibitor 2A (p16(INK4a)) gene |
| CEBPA | chr19:33790842-33793430 | CCAAT/enhancer binding protein (C/EBP), alpha |
| CHEK1 | chr11:125496312-125525639 | CHK1 checkpoint homolog (S. pombe) |
| CHEK2 | chr22:29083731-29137822 | CHK2 checkpoint homolog |
| COL14A1 | chr8:121137352-121384266 | collagen, type XIV, alpha 1 |
| CPAMD8 | chr19:17003763-17137625 | C3 and PZP-like, alpha-2-macroglobulin domain containing 8 |
| CREBBP | chr16:3775058-3930121 | CREB binding protein (CBP) |
| CRIPAK | chr4:1385340-1389782 | cysteine-rich PAK1 inhibitor |
| CSF1R | chr5:149432855-149492935 | colony stimulating factor 1 receptor |
| CSMD1 | chr8:2792876-4852328 | CUB and Sushi muliple domain 1 |
| CSMD2 | chr1:33979609-34630875 | CUB and Sushi multiple domains 2 |
| CSMD3 | chr8:113235161-114449242 | CUB and Sushi muliple domain 3 |
| CTNNB1 | chr3:41240942-41281939 | catenin (cadherin-associated protein), beta 1 |
| CYLD | chr16:50775961-50835846 | familial cylindromatosis gene |
| CYP2C19 | chr10:96522463-96612670 | cytochrome P450, family 2, subfamily C, polypeptide 19 |
| DAXX | chr6:33,286,336-33,290,793 | death-domain associated protein |
| DDR1 | chr6:30856465-30867931 | discoidin domain receptor tyrosine kinase 1 |
| DDR2 | chr1:162602228-162750237 | discoidin domain receptor tyrosine kinase 2 |
| DNMT3A | chr2:25455846-25564774 | DNA (cytosine-5-)-methyltransferase 3 alpha |
| EGFR | chr7:55086725-55275030 | epidermal growth factor receptor |
| ELN | chr7:73442427-73484234 | elastin |
| EML4 | chr2:42,396,490-42,559,686 | echinoderm microtubule associated protein like 4 |
| EP300 | chr22:41488614-41576080 | 300 kd E1A-Binding protein gene |
| EPHA3 | chr3:89156674-89531282 | EPH receptor A3 |
| ERBB2 | chr17:37856254-37884914 | v-erb-b2 erythroblastic leukemia viral oncogene homolog 2 |
| ERBB3 | chr12:56,473,892-56,497,127 | v-erb-b2 erythoblastic lekemia viral oncogene homolog 3 (avian) |
| ERCC3 | chr2:128014866-128051752 | excision repair cross-complementing rodent repair deficiency, compl group 3 |
| ERCC4 | chr16:14014014-14046205 | excision repair cross-complementing rodent repair deficiency, compl group 4 |
| ERCC5 | chr13:103459496-103524748 | excision repair cross-complementing rodent repair deficiency, comp group 6 |
| ESR1 | chr6:152163859-152424408 | estrogen Receptor 1 |
| ETV5 | chr3:185764108-185826901 | ets variant 5 |
| EZH2 | chr7:148504475-148581414 | cer of zeste homolog 2 (Drosophila) |
| FAM123B | chrX:63404998-63425624 | family with sequence similarity 123B (FAM123B) |
| FAM135B | chr8:139142268-139509065 | family with sequence similarity 135, member B |
| FAT3 | chr11:92,085,262-92,629,633 | fat tumor suppressor 3 |
| FBXW7 | chr4:153242411-153456185 | F-box and WD-40 domain protein 7 (archipelago homolog, Drosophila) |
| FGFR1 | chr8:38268657-38326352 | FGFR1 oncogene partner (FOP) |
| FGFR2 | chr10:123237845-123357972 | fibroblast growth factor receptor 2 |
| FGFR3 | chr4:1795039-1810599 | fibroblast growth factor receptor 3 |
| FGFR4 | chr5:176513921-176525124 | fibroblast growth factor receptor 4 |
| FLG | chr1:152274651-152297679 | filaggrin |
| FLT1 | chr13:28874483-29069265 | fms-related tyrosine kinase 1 (VEGF/vascular permeability factor receptor) |
| FLT3 | chr13:28577412-28674729 | fms-related tyrosine kinase 3 |
| FLT4 | chr5:180028507-180076624 | fms-related tyrosine kinase 4 |
| FOXL2 | chr3:138,663,067-138,665,982 | forkhead box L2 |
| GABRA6 | chr5:161112658-161129598 | GABA A receptor, alpha 6 |
| GABRB3 | chr15:26788695-27018251 | GABA A1 receptor, beta 3 |
| GATA1 | chrX:48,644,982-48,652,715 | GATA binding protein 3 |
| GATA3 | chr10:8096667-8117162 | guanine monphosphate synthetase |
| GNA11 | chr19:3094408-3121452 | guanine nucleotide binding protein (G protein), alpha 11 (Gq class) |
| GNAQ | chr9:80335200-80646192 | guanine nucleotide binding protein (G protein), q polypeptide |
| GNAS | chr20:57414795-57486249 | guanine nucleotide binding protein (G protein), alpha stimulating activ polyp 1 |
| HDAC9 | chr7:18535885-19036984 | histone deacetylase 9 |
| HEATR7B2 | chr5:40998123-41071444 | HEAT repeat family member 7B2 |
| HGF | chr7:81331445-81399452 | hepatocyte growth factor |
| HMCN1 | chr1:185703683-186160085 | hemicentin 1 |
| HNF1A | chr12:121416549-121440312 | transcription factor 1, hepatic (HNF1) |
| HNF1B | chr17:36046435-36105096 | HNF1 homeobox B |
| HRAS | chr11:532243-535550 | v-Ha-ras Harvey rat sarcoma viral oncogene homolog |
| HYDIN | chr16:70841290-71264569 | hydrocephalus inducing homolog |
| IDH1 | chr2:209100954-209119806 | isocitrate dehydrogenase 1 (NADP+), soluble |
| IDH2 | chr15:90627214-90645708 | isocitrate dehydrogenase 2 (NADP+), soluble |
| IGF1R | chr15:99192761-99507758 | insulin-like growth factor 1 receptor |
| IKZF1 | chr7:50344378-50472796 | interleukin 21 receptor |
| IL6R | chr1:154377669-154440188 | interleukin 6 receptor |
| IRS1 | chr2:227596034-227663506 | insulin receptor substrate 1 |
| ITGA4 | chr2:182321619-182402466 | integrin alpha 4 (antigen CD49D subunit of VLA-4 receptor) |
| JAK1 | chr1:65298906-65432187 | Janus kinase 1 |
| JAK2 | chr9:4985245-5128182 | Janus kinase 3 |
| JAK3 | chr19:17935595-17958841 | Janus kinase 3 |
| KCNB2 | chr8:73449626-73850582 | potassium voltage-gated channel, Shab-related subfamily, member 2 |
| KDM6A | chrX:44732423-44971843 | vascular endothelial growth factor receptor 2 |
| KDR | chr4:55944427-55991762 | vascular endothelial growth factor receptor 2 |
| KIT | chr4:55524095-55606879 | kallikrein-related peptidase 2 |
| KRAS | chr12:25358180-25403854 | v-Ki-ras2 Kirsten rat sarcoma 2 viral oncogene homolog |
| LAMA1 | chr18:6941888-7117813 | laminin, alpha 1 |
| LPHN3 | chr4:62362839-62938167 | latrophilin 3 |
| LRP1 | chr12:57,522,282-57,607,123 | low density lipoprotein receptor-related protein 1B |
| LRP1B | chr2:140988996-142889270 | low density lipoprotein receptor-related protein 2 |
| LRP2 | chr2:169983620-170219122 | low density lipoprotein receptor-related protein 3 |
| MAP2K1 | chr15:66679211-66783881 | mitogen-activated protein kinase kinase 1 |
| MAP2K4 | chr17:11924135-12047050 | mitogen-activated protein kinase kinase 4 |
| MAP3K1 | chr5:56110900-56191976 | mitogen-activated protein kinase kinase kinase 1 |
| MAP3K4 | chr6:161412822-161538416 | mitogen-activated protein kinase kinase kinase 4 |
| MDN1 | chr6:90353231-90529442 | MDN1, midasin homolog |
| MECOM | chr3:168801287-169381563 | MDS1 and EVI1 complex locus |
| MEN1 | chr11:64570996-64578188 | multiple endocrine neoplasia type 1 gene |
| MET | chr7:116312459-116438439 | met proto-oncogene (hepatocyte growth factor receptor) |
| MITF | chr3:69788633-70017486 | microphthalmia-associated transcription factor |
| MLH1 | chr3:37034979-37092335 | E.coli MutL homolog gene |
| MLL2 | chr12:49412762-49449107 | myeloid/lymphoid or mixed-lineage leukemia 2 |
| MLL3 | chr7:151832012-152133090 | myeloid/lymphoid or mixed-lineage leukemia 3 |
| MPL | chr1:43803475-43820134 | myeloproliferative leukemia virus oncogene |
| MSH2 | chr2:47630263-47710360 | mutS homolog 2 (E. coli) |
| MSH6 | chr2:48,010,221-48,034,084 | mutS homolog 6 (E. coli) |
| MTOR | chr1:11166589-11322608 | mammalian target of rapamycin complex 1 |
| MYD88 | chr3:38179969-38184510 | myeloid differentiation primary response gene (88) |
| NAV3 | chr12:78225069-78606788 | neuron navigator 3 |
| NCOR1 | chr17:15935259-16118845 | nuclear receptor corepressor 1 |
| NF1 | chr17:29,421,995-29,704,694 | neurofibromatosis type 1 gene |
| NF2 | chr22:29999545-30094583 | neurofibromatosis type 2 gene |
| NFKB2 | chr10:104154339-104162280 | nuclear factor of kappa light polypeptide gene enhancer in B-cells 2 |
| NOTCH1 | chr9:139388897-139440238 | Notch homolog 1, translocation-associated (Drosophila) (TAN1) |
| NOTCH2 | chr1:120454178-120612276 | Notch homolog 2 |
| NOTCH3 | chr19:15270445-15311792 | notch 3 |
| NOTCH4 | chr6:32162621-32191844 | notch4 |
| NPM1 | chr5:170814798-170837887 | nucleophosmin (nucleolar phosphoprotein B23, numatrin) |
| NRAS | chr1:115247079-115259515 | neuroblastoma RAS viral (v-ras) oncogene homolog |
| NSD1 | chr5:176560833-176727213 | nuclear receptor binding SET domain protein 1 |
| PALB2 | chr16:23614483-23652678 | partner and localizer of BRCA2 |
| PAPPA2 | chr1:176432307-176811968 | pappalysin 2 |
| PAX5 | chr9:36838531-37034476 | paired box gene 5 (B-cell lineage specific activator protein) |
| PBRM1 | chr3:52,579,368-52,713,739 | polybromo 1 |
| PCDH15 | chr10:55580860-56561051 | protocadherin 1 |
| PCLO | chr7:82383321-82792197 | piccolo (presynaptic cytomatrix protein) |
| PDGFRA | chr4:55095264-55164411 | platelet-derived growth factor, alpha-receptor |
| PDGFRB | chr5:149493403-149535422 | platelet-derived growth factor receptor, beta polypeptide |
| PIK3CA | chr3:178866311-178952495 | phosphoinositide-3-kinase, catalytic, alpha polypeptide |
| PIK3CG | chr7:106505924-106547585 | phosphoinositide-3-kinase, catalytic, gamma polypeptide |
| PIK3R1 | chr5:67522118-67597647 | phosphoinositide-3-kinase, regulatory subunit 1 (alpha) |
| PIKFYVE | chr2:209130991-209223474 | protein phosphatase 2A activator, regulatory subunit 4 |
| PKHD1 | chr6:51480145-51952423 | polycystic kidney and hepatic disease 1 (autosomal recessive) |
| PKHD1L1 | chr8:110374706-110543499 | polycystic kidney and hepatic disease 1 (autosomal recessive)-like 1 |
| PPP1R3A | chr7:113516882-113559082 | protein phosphatase 1, regulatory (inhibitor) subunit 3A |
| PPP2R1A | chr19:52693191-52729670 | protein phosphatase 2, regulatory subunit A, alpha |
| PPP2R4 | chr9:131873244-131911223 | protein phosphatase 2A activator, regulatory subunit 4 |
| PRDM1 | chr6:106534195-106557814 | PR domain containing 1, with ZNF domain |
| PRSS1 | chr7:142457319-142460927 | protease, serine, 1 (trypsin 1) |
| PTCH1 | chr9:98205266-98270831 | Homolog of Drosophila Patched gene1 |
| PTEN | chr10:89623195-89728531 | phosphatase and tensin homolog gene |
| PTK2 | chr8:141668502-142011332 | PTK2 protein tyrosine kinase 2 |
| PTPN11 | chr12:112856536-112947716 | protein tyrosine phosphatase, non-receptor type 11 |
| RAD51 | chr15:40987327-41024354 | RAD51 homolog |
| RAF1 | chr3:12625102-12705700 | v-raf-1 murine leukemia viral oncogene homolog 1 |
| RB1 | chr13:48877883-49056024 | retinoblastoma gene |
| RELN | chr7:103112233-103629963 | reelin |
| RET | chr10:43572517-43625795 | ret proto-oncogene |
| RIMS2 | chr8:104512976-105265451 | regulating synaptic membrane exocytosis 2 |
| RNF213 | chr17:78313726-78370078 | ring finger protein 213 |
| RUNX1 | chr21:36160099-36421595 | runt-related transcription factor 1 (AML1) |
| RUNX1T1 | chr8:92971152-93088365 | runt-related transcription factor 1 |
| RYR2 | chr1:237205702-237997288 | regulatory factor X, 2 (influences HLA class II expression) |
| SETD2 | chr3:47057900-47205467 | SET domain containing 2 |
| SMAD4 | chr18:48556583-48611409 | SMAD family member 4 |
| SMARCA4 | chr19:11071598-11172959 | SWI/SNF related, matrix assoc, actin dep reg of chrom, subfamily a, member 4 |
| SMARCB1 | chr22:24129150-24176704 | SWI/SNF related, matrix assoc, actin dep reg of chrom, subfamily b, member 1 |
| SMO | chr7:128828713-128853383 | smoothened homolog (Drosophila) |
| SOS1 | chr2:39208692-39347604 | son of sevenless homolog 1 |
| SPEN | chr1:16174359-16266950 | spen homolog, transcriptional regulator |
| SPOP | chr17:47676248-47755525 | speckle-type POZ protein |
| SPTA1 | chr1:158580496-158656506 | spectrin alpha, erythrocytic 1 |
| STK11 | chr19:1205798-1228434 | serine/threonine kinase 11 gene (LKB1) |
| SYK | chr9:93564012-93660833 | spleen tyrosine kinase |
| SYNE1 | chr6:152442823-152958534 | spectrin repeat containing, nuclear envelope 1 |
| SYNE2 | chr14:64319683-64693165 | spectrin repeat containing, nuclear envelope 2 |
| TBC1D4 | chr13:75858809-76056250 | TBC1 domain family, member 4 |
| TET2 | chr4:106067943-106200958 | tet oncogene family member 2 |
| TGFb1 | chr19:41836651-41859816 | transforming growth factor, beta 1 |
| TGFBR2 | chr3:30647994-30735631 | transforming growth factor, beta receptor II |
| TNFAIP3 | chr6:138188581-138204445 | tumor necrosis factor, alpha-induced protein 3 |
| TOP1 | chr20:39657462-39753124 | topoisomerase (DNA) I |
| TOP2A | chr17:38544798-38574169 | topoisomerase II |
| TP53 | chr17:7571720-7590863 | tumor protein p53 |
| TSC1 | chr9:135766735-135820020 | tuberous sclerosis 1 |
| TSC2 | chr16:2097990-2138712 | tuberous sclerosis 2 |
| TSHR | chr14:81421869-81612646 | thyroid stimulating hormone receptor |
| USH2A | chr1:215796236-216596738 | usher syndrome 2A |
| VHL | chr3:10183319-10193744 | von Hippel-Lindau syndrome gene |
| WHSC1 | chr4:1873123-1983933 | Wolf-Hirschhorn syndrome candidate 1 |
| WT1 | chr11:32409325-32457087 | Wilms tumour 1 gene |
| ZNF238 | chr1:244214561-244220776 | zinc finger protein 238 |
| ZNF536 | chr19:30863328-31048965 | zinc finger protein 536 |
